# Editorial: Grand Challenges in Pharmaceutical Medicine: Competencies and Ethics in Medicines Development

**DOI:** 10.3389/fphar.2021.666406

**Published:** 2021-05-13

**Authors:** Honorio Silva, Peter Stonier, Sandor Kerpel-Fronius, Dominique Dubois

**Affiliations:** ^1^ IFAPP Academy, New York, NY, United States; ^2^ Institute of Pharmaceutical Science King’s College, London, United Kingdom; ^3^ Department of Pharmacology and Pharmacotherapy, Faculty of Medicine, Semmelweis University, Budapest, Hungary; ^4^ Université Libre de Bruxelles, Brussels, Belgium

**Keywords:** professional development, postgraduate education, pharmaceutical medicine, curriculum development, competency based learning

## Introduction

This Research Topic represents a collaboration between the International Federation of Associations of Pharmaceutical Physicians and Pharmaceutical Medicine (IFAPP) and Frontiers in Pharmaceutical Medicine and Outcomes Research aimed to create further awareness of Pharmaceutical Medicine (PM) as a profession and meet the new challenges of medicines development.

## Why?

The advancement in biomedical sciences extended the concept of medical products to including biological agents, gene and cell therapies as well as drug-medical device combinations. The development and application of these new products can be efficiently done only in complex, multidisciplinary teams combining the know-how of pharmaceutical physicians, clinical investigators, basic and applied bio-medical scientists and other non-medically qualified professionals.

## How?

This Research Topic covers 11 articles focusing on the evolving challenges in medicines development as related to the standards for performing with competence and the application of ethical principles while working in the pharmaceutical industry, academia, research sites and regulatory agencies.

The circumstances related to the COVID-19 pandemic underscores the role of the biopharmaceutical industry as a key link between basic biomedical discovery and the emergence of novel medicines that prolong or improve life. Medicines development can be defined as an open system involving patients, investigators and associated staff, regulators, sponsors, research sites, etc. as components interconnected through a series of processes aimed to bring effective and safe medicines into the market and maintain them. Because of the above a *systems approach integrating research into healthcare system*s has been proposed to overcome the current barriers to a cost/effective cooperative process and appropriate management of the risks involved (Meadows, 2008; Johnson et al, 2014; Silva et al, 2015).

## The Challenges

However, a host of challenges confront healthcare authorities worldwide. The challenge is particularly great in therapeutic areas where, despite significant medical need and economic impact, the technical challenges and commercial risk of development serve as disincentives to sponsors. Currently the development and approval of new active substances, with its disproportionate focus on oncology and rare diseases is not in alignment with health care needs in most geographic regions. The origins of this misalignment and approaches to overcome this situation are discussed (Milne and Kaitin) with an urgent call to address these disparities using a multi-stakeholder approach and building consensus for change.

Clinical trials constitute the largest single component in medicines development, representing nearly 40% of the R&D expenses of major companies. However, there is broad agreement that the current clinical trial system is inefficient. The biopharmaceutical industry, governments and regulatory agencies, academic researchers, the medical community and the media should work collaboratively to fill the gaps and create efficient clinical trial networks and trial designs.

The lack of an adequately sized and appropriately trained multi-professional workforce both in the industry-related and the academic clinical research field is also a significant part of the situation. The root of the problem resides in the lack of proper education in clinical research and pharmaceutical medicine at the undergraduate and postgraduate levels across academic institutions worldwide. Only a few universities are directly involved in this process and thus professionals joining the industry usually gain competence through on-the-job training. The outcomes of an IFAPP sponsored international survey aimed to assess the self-perception of competence, education and training needs among biomedical professionals serving in the various functions in the pharmaceutical industry are described (Imamura et al.) indicating low and variable levels of perceived competence for the various domains regardless of the seniority in the job. Similar results were reported among individuals involved in clinical research (Sonstein et al., 2016) underscoring the need for proper education and training (E&T) worldwide.

The evolution of postgraduate vocational E&T in pharmaceutical medicine along with the development of the full set of core competencies (knowledge, skills, behaviors and attitudes to perform a task) for pharmaceutical physicians and drug development scientists within the competence framework of seven domains are now established (Stonier et al.). Core Competencies in Clinical Research have also been identified and proposed as a model for E&T and improving the quality and accountability for specific functions involved in the drug development process (Sonstein and Jones) including the challenges for implementation and lessons learned.

Many of the disruptive forces affecting the healthcare industry today are also impacting education. The increasing voice of the patient and the rise of patient engagement in drug development are mirrored by the increasing student voice and student focus on education. The process for curriculum transformation from didactic to competency based programs in Pharmaceutical Medicine in Australia is thoroughly described (Chisholm) whereas the process of adoption of the scope of the above Framework to reflect such roles in academic institutions or regulatory bodies in Switzerland is part of the lessons learned (Schnetzler et al.)*.*

## Use of Competency Based Education

There is a growing consensus of the role of vocational training to gain competence (UNESCO, 2019). Specific vocational programs in medicines development have been implemented in the UK and Ireland for several years leading to a national medical board certification in Pharmaceutical Medicine. IFAPP and PharmaTrain developed the vocational Specialist in Medicines Development Program sponsored by the IMI. The outcomes of pilot experiences in Italy and Japan are encouraging (Criscuolo et al.) with recommendations to all other countries and institutions which may consider establishing this program**.**

Regulatory Affairs professionals play pivotal roles to ensuring healthcare products adhere to regulations and in gaining regulatory approvals for product manufacture and sales. Although they perform complicated work connected to the entire product lifecycle, only 14% of regulatory professionals come to the field with a degree related to the work (Regulatory Affairs Professional Society, 2018) and more than half are involved in regulatory work as a second career. Professional Associations are key in making efforts to develop and align competencies for regulatory professionals and create a competent global regulatory workforce (Bridges).

## Ethical Considerations

The complexity of developing and applying increasingly sophisticated new medicinal products has led to the participation of many medical and non-medically qualified scientists in multidisciplinary non-clinical and clinical drug development teams worldwide. Revising the IFAPP International Code of Ethical Conduct for Pharmaceutical Physicians written in 2003, the Ethics Working Group prepared the IFAPP International Ethics Framework (Kerpel-Fronius et al.) with the intention to provide recommendations to both professional groups to make joint ethical decisions during various situations occurring during clinical trials. Mutual trust and respect between the various experts is emphasized as the basis of effective multi-professional team work.

These revised recommendations add to the list of Codes of Practice for pharmaceutical physicians prepared by professional organizations like the Faculty of Pharmaceutical Medicine, CIOMS and the World Medical Association. Jointly they provide clear and detailed guidance for correct behavior of pharmaceutical medicine experts in specific research situations (Morris et al.)*.*

An alignment of the Declarations of Helsinki with that of the Declaration of Taipei is recommended for the better protection of both biological materials and data derived from clinical studies when their secondary use is intended. Furthermore, it is emphasized that any future plan for data and/or material sharing should be explained in the protocol, signed by the research participants and should be made publicly available (Kurihara et al.).

This Research Topic intended to create further awareness of the complex set of competencies and ethical considerations required for clinical drug development and the need to foster education and training at the undergraduate, postgraduate and continuing professional development levels to ensure the pharmaceutical industry is fledged with competent professionals able to bring better and valuable medicines to the market place and contribute to leveraged health in their communities.
